# Supplementary material for: Tunnel field-effect transistors for sensitive terahertz detection
Source: Nat Commun. 2021 Jan 22;12:543. doi: 10.1038/s41467-020-20721-z (PMC7822863; doi:10.1038/s41467-020-20721-z)
Supplement: Supplementary file 1 — Supplementary Information [file 41467_2020_20721_MOESM1_ESM.pdf]

SUPPLEMENTARY INFORMATION

for

Tunnel field-effect transistors for sensitive terahertz detection

I. Gayduchenko, S.G. Xu, G. Alymov, M. Moskotin, I. Tretyakov, T. Taniguchi, K. Watanabe, G. Goltsman, A.K. Geim, G. Fedorov, D. Svintsov, and D.A. Bandurin.

Supplementary Note 1: Further examples of tunnel-enabled photoresponse

Supplementary Figure 1 shows further examples of our tunnel detector responsivity R_v as a function of V_tg recorded in response to $f = 0.13$ THz radiation for varying V_bg. For all $V_\mathrm{bg} \neq 0$, $R_\mathrm{v}(V_\mathrm{tg})$ dependencies are highly asymmetric. With increasing V_bg, R_v is increasing and for $V_\mathrm{bg} = 2.6$ V reaches 4.5 kV/W overcoming zero V_bg value by more than an order of magnitude. A similar behaviour was observed if the polarity of V_bg is reversed (blue curve in Supplementary Figure 1). These observations highlights a drastic difference between the field-effect-enabled intraband (black curve) rectification and its interband tunneling counterpart (all other curves).

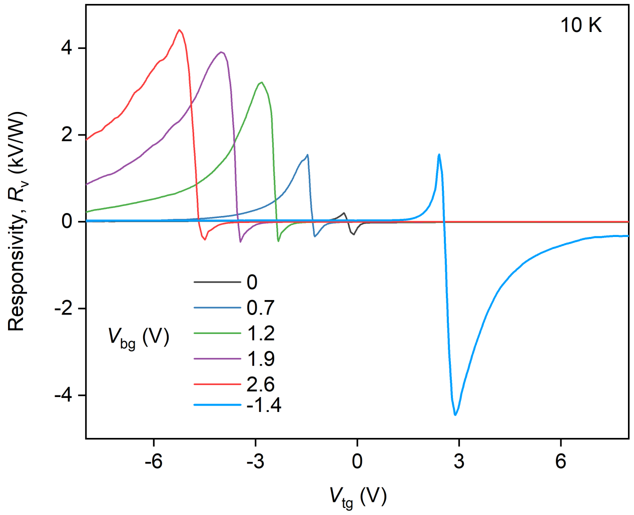

Supplementary Figure 1. **Tunneling-enabled THz detection.** R_v as a function of V_tg for given V_bg recorded in response to 0.13 THz radiation. $T = 10$ K.

Supplementary Note 2: Frequency dependence of tunnelling-enabled photoresponse

We have also studied the response of our detectors at higher frequency and found consistent tunnelling-enabled highly-asymmetric behaviour when the top and bottom gates are biased with opposite polarity. Examples of $R_v(V_{tg})$ are shown in Supplementary Figure 2a for two characteristic f from sub-THz and THz domains. Note, due to the limitation of our measurements (See Methods) we only present a relative comparison between $R_v(V_{tg})$ recorded at $f = 0.13$ THz and $f = 2$ THz. However, our modelling which provides remarkable agreement with experiment at $f = 0.13$ THz predicts that TFET detectors are expected to perform equivalently well at both sub-THz and THz frequencies as we show in Supplementary Figures 2b,c.

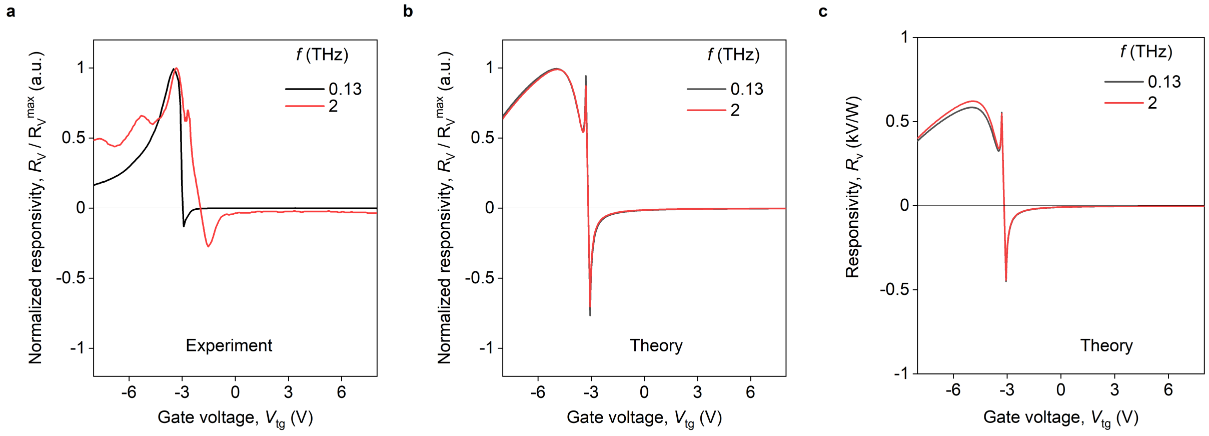

Supplementary Figure 2. **Frequency dependence of tunnelling-assisted THz detection. a** R_v as a function of V_{tg} for $V_{bg} = 1.2$ V obtained under illumination with THz radiation of given frequency. The data normalized to their maximum value. Peaks in R_v correspond to the excitation of plasmon-resonances in the detector channel[S1]. **b, c** Theoretical $R_v(V_{tg})$ dependencies for given f: as-calculated (**c**) and normalized to their maximum value (**b**).

Supplementary Note 3: Comparison with existing technology

In Supplementary Figure 3 we compare the performance of our tunnel device with other THz detectors and rectifiers; some of them are available on the market (underlined labels). To this end, we plot their noise equivalent power (NEP) versus temperature, T, at which they operate. The comparison is made for the frequency range $0.1 - 2$ THz and for the NEP calculated via extrinsic responsivity, i.e. which takes into account the full power delivered to the device. The devices of different types are compared: cooled superconducting bolometers[S2,S3], cooled semiconducting bolometers[S4–S6], kinetic inductance sensors[S7,S8], cooled quantum dot devices[S9,S10], as well as transistor-based detectors[S1,S11–S18], Schottky diodes[S19–S21], and heterostructure backward diodes[S22,S23]. One of the primary tasks for the next-generation THz technology is to produce low-NEP sensors operating at elevated temperatures as indicated by the yellow shaded area in Supplementary Figure 3. However, whereas the cooled devices feature exceptionally low NEP, room-T devices are usually characterized by much higher NEP. Our BLG TFET offers a compromise to this enquiry: it features relatively low NEP and operates above liquid helium T. Furthermore, our model suggests that TFETs with optimized parameters can feature even lower NEP at room temperature (magenta star in Supplementary Figure 3) and thus offer a route to the next-generation THz technology. The details are given in Supplementary Note 5.

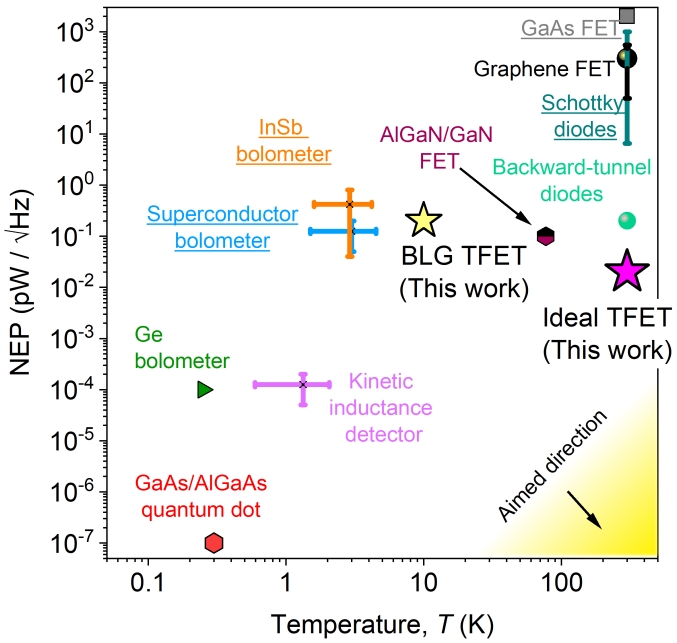

Supplementary Figure 3. Overview of THz detectors. NEP for THz detectors of various types plotted against the temperature at which they operate. Vertical error bars represent the spread of the detectors' performance over the frequency range $0.1 - 2$ THz. Horizontal error bars show the temperature range at which the detectors operate. Underlined labels denote commercial technology.

Supplementary Note 4: Theoretical model of a BLG TFET photodetector

Supplementary Note 4.1: Modelling of tunneling-assisted THz detection

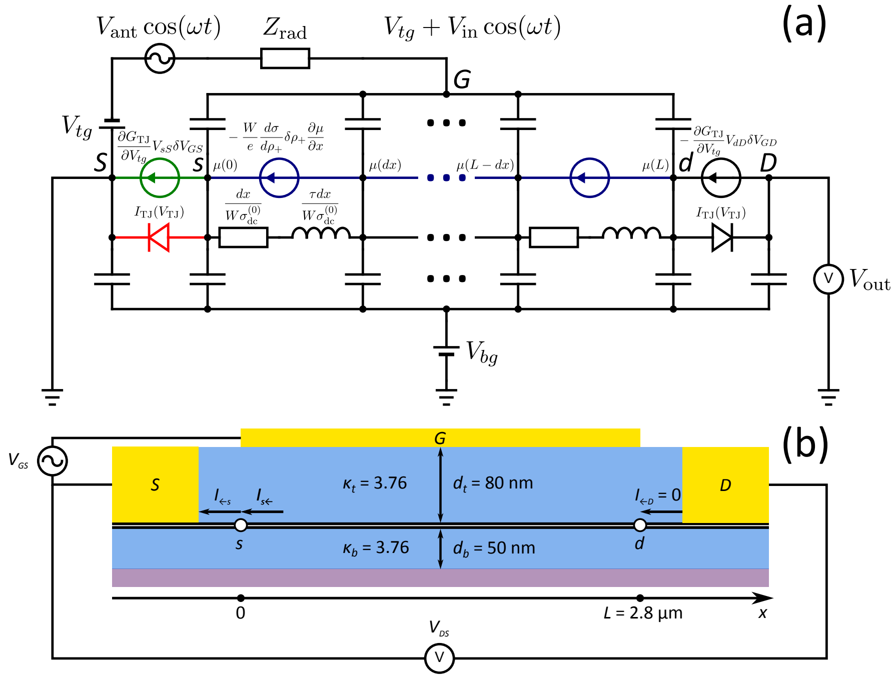

Supplementary Figure 4. **Equivalent circuit. a,** Detailed equivalent circuit of a TFET-based detector. Different colors show the origins of different contributions to responsivity: nonlinear current-voltage characteristic of the tunnel junction between source and channel (red), its gate-controlled conductance (represented as an equivalent current source, green), and the gate-controlled channel conductance (represented as a distributed current source, blue). **b,** Schematic view of our photodetector showing some of the notation used throughout the Supplementary Information.

In this section, we derive a general expression for the responsivity of a TFET. The relevant circuit is shown in Supplementary Figure 4a. We will treat the TFET as if it was single-gated, since the bottom gate is held at a constant potential and its only function is to open a bandgap in BLG.

A TFET consists of two rectification units: a tunnel junction between the source and the channel, and the channel itself. (The drain tunnel junction is effectively excluded from the circuit by the zero drain current assumption, at least if the junction is too short to accommodate any spatial inhomogenities of the current.)

When a small ac voltage $V_{\rm in}\cos(\omega t)$ is applied between the gate and source, it induces voltages and currents in different parts of the detector, having the general form $(\delta V, \delta I)(t) = \mathrm{Re}\left((V,I)^{(1)}e^{-i\omega t}\right) + (V,I)^{(2)} + \ldots \cdot e^{\pm 2i\omega t} + o(V_{\rm in}^2)$, where we are interested in the first-order and dc second-order components.

We use a non-distributed model for the source junction, meaning current can flow through the junction only in presence of a nonzero voltage drop across the junction and not solely under the action of ac gate voltage. Keeping this is mind, the second-order expansion of its current-voltage characteristic $I_{\leftarrow s}(V_{sS}, V_{GS})$ reads

$$
\begin{aligned}
I_{\leftarrow s}^{(1)} &= G_S(\omega)V_{sS}^{(1)}, \\
I_{\leftarrow s}^{(2)} &= G_{S,\rm dc}\left[V_{sS}^{(2)} - R_{\rm TJ,\it i}\frac{\left|V_{sS}^{(1)}\right|^2}{2} - R_{\rm TG,\it i}\frac{\mathrm{Re}\left(V_{GS}^{(1)}V_{sS}^{(1)*}\right)}{2}\right],
\end{aligned} \tag{S1}
$$

where $G_{S,\rm dc}$ and $G_S(\omega)$ are the dc and ac conductance of the junction, $R_{\mathrm{TJ},i}$ is the intrinsic tunnel junction responsivity, and $R_{\mathrm{TG},i}$ is the intrinsic "tunnel-gate" responsivity.

When writing similar expressions for the current $I_{s\leftarrow}$ flowing to the source from the channel, we make advantage of the linear dependence between $V_{ds}^{(1)}$ and $V_{Gs}^{(1)}$ arising from zero drain current condition, and use only $V_{Gs}^{(1)}$ and $V_{ds}^{(2)}$ as independent variables (remember that dc gate voltage $V_{Gs}^{(2)}$ does not produce any current by itself):

$$
\begin{aligned}
I_{s\leftarrow}^{(1)} &= \tilde{G}_{\mathrm{ch}}(\omega) V_{Gs}^{(1)}, \\
I_{s\leftarrow}^{(2)} &= G_{\mathrm{ch,dc}} \left[V_{ds}^{(2)} - R_{\mathrm{ch},i} \frac{\left| V_{Gs}^{(1)} \right|^2}{2} \right],
\end{aligned} \tag{S2}
$$

where $G_{\mathrm{ch,dc}}$ is the dc channel conductance, $\tilde{G}_{\mathrm{ch}}(\omega) \equiv (\partial I_{s\leftarrow}(\omega)/\partial V_{Gs}(\omega))\,|_{I_{\leftarrow d}=0}$ is the ac channel conductance measured between source and gate, and $R_{\mathrm{ch},i}$ is the intrinsic channel responsivity.

From continuity of current, $I_{\leftarrow s}^{(1)} = I_{s\leftarrow}^{(1)}$ and $I_{\leftarrow s}^{(2)} = I_{s\leftarrow}^{(2)} = I_{\leftarrow D}^{(2)} = 0$, we find that the ac voltage $V_{GS}^{(1)} \equiv V_{\mathrm{in}}$ applied between the gate and source is divided into voltage $V_{sS}^{(1)}$ at the source tunnel junction and voltage $V_{Gs}^{(1)}$ between the gate and the beginning of the channel:

$$
\begin{aligned}
V_{sS}^{(1)} &= \frac{\tilde{G}_{\mathrm{ch}}(\omega)}{G_S(\omega) + \tilde{G}_{\mathrm{ch}}(\omega)} V_{\mathrm{in}}, \\
V_{Gs}^{(1)} &= \frac{G_S(\omega)}{G_S(\omega) + \tilde{G}_{\mathrm{ch}}(\omega)} V_{\mathrm{in}},
\end{aligned} \tag{S3}
$$

which are subsequently rectified by the tunnel junction and the channel:

$$
\begin{aligned}
V_{sS}^{(2)} &= R_{\mathrm{TJ},i} \frac{\left| V_{sS}^{(1)} \right|^2}{2} + R_{\mathrm{TG},i} \frac{\mathrm{Re}\left(V_{GS}^{(1)} V_{sS}^{(1)*} \right)}{2}, \\
V_{ds}^{(2)} &= R_{\mathrm{ch},i} \frac{\left| V_{Gs}^{(1)} \right|^2}{2}.
\end{aligned} \tag{S4}
$$

These rectified voltages sum together to yield the output voltage $V_{DS}^{(2)} \equiv V_{\mathrm{out}}$ of the photodetector (remember that the voltage at the drain junction $V_{Dd}^{(2)} = 0$ because of zero drain current). Total responsivity of the TFET is given by the sum of tunnel junction responsivity, coming from the nonlinear current-voltage characteristic of the source tunnel junction, tunnel-gate responsivity, coming from resistive self-mixing in the gate-controlled source tunnel junction, and channel responsivity, coming from resistive self-mixing in the channel:

$$
\begin{aligned}
R_{\mathrm{TFET}} &\equiv \frac{V_{\mathrm{out}}}{V_{\mathrm{in}}^2/2} \equiv \frac{V_{DS}^{(2)}}{\left(V_{GS}^{(1)} \right)^2 /2} = R_{\mathrm{TJ}} + R_{\mathrm{TG}} + R_{\mathrm{ch}}, \\
R_{\mathrm{TJ}} &\equiv \left| \frac{\tilde{G}_{\mathrm{ch}}(\omega)}{G_S(\omega) + \tilde{G}_{\mathrm{ch}}(\omega)} \right|^2 R_{\mathrm{TJ},i}, \\
R_{\mathrm{TG}} &\equiv \mathrm{Re}\, \frac{\tilde{G}_{\mathrm{ch}}(\omega)}{G_S(\omega) + \tilde{G}_{\mathrm{ch}}(\omega)} R_{\mathrm{TG},i}, \\
R_{\mathrm{ch}} &\equiv \left| \frac{G_S(\omega)}{G_S(\omega) + \tilde{G}_{\mathrm{ch}}(\omega)} \right|^2 R_{\mathrm{ch},i}.
\end{aligned} \tag{S5}
$$

We will neglect the frequency dependence of the tunnel junction current-voltage characteristic. With this assumption, intrinsic tunnel junction and tunnel-gate responsivities are given by the logarithmic derivatives of the junction conductance with respect to appropriate voltages:

$$
\begin{aligned}
R_{\mathrm{TJ},i} &= -\frac{1}{2} \left(\frac{\partial \ln G_S}{\partial V_{sS}} \right)_{V_{GS}}, \\
R_{\mathrm{TG},i} &= -\left(\frac{\partial \ln G_S}{\partial V_{GS}} \right)_{V_{sS}}.
\end{aligned} \tag{S6}
$$

Due to the distributed nature of the channel, its current-voltage characteristics are inherently frequency-dependent. Nevertheless, the intrinsic channel responsivity can also be expressed in terms of the logarithmic derivative of its dc conductance, see Supplementary Note 4.6:

$$R_{\mathrm{ch},i} \approx -\frac{1}{2}\frac{d_b}{d_t+d_b}\left(\frac{\partial \ln G_{\mathrm{ch,dc}}}{\partial V_{Gs}}\right)_{V_{ds}=0}. \tag{S7}$$

A similar expression was originally derived in Ref. S24 for a single-gated FET. The extra prefactor represents the gate voltage division in a double-gated structure with top and bottom gate dielectrics of thicknesses d_t, d_b.

TFET responsivity (S5) describes its response to the ac voltage at the gate, while the experimentally measured photodetector responsivity R_{v} describes response to the power P_{in} incident on the antenna. The relation between these responsivities can be obtained by considering the complete circuit of the photodetector, including the antenna radiation resistance $Z_{\mathrm{rad}}(\omega)$ (Supplementary Figure 4a). Assuming the incident radiation is focused within the antenna's effective aperture, the incident power can be converted into the effective voltage $V_{\mathrm{ant}} = \sqrt{8Z_{\mathrm{rad}}(\omega)P_{\mathrm{in}}}$[S25], which is divided between $Z_{\mathrm{rad}}(\omega)$ and the TFET gate-to-source ac impedance

$$Z_{\mathrm{GS}}(\omega) = G_S^{-1} + \tilde{G}_{\mathrm{ch}}^{-1}(\omega), \tag{S8}$$

yielding

$$R_{\mathrm{v}} \equiv \frac{V_{\mathrm{out}}}{P_{\mathrm{in}}} = 8Z_{\mathrm{rad}}(\omega)\frac{V_{\mathrm{out}}}{|V_{\mathrm{ant}}|^2} = 4Z_{\mathrm{rad}}(\omega)\left|\frac{Z_{\mathrm{GS}}(\omega)}{Z_{\mathrm{GS}}(\omega)+Z_{\mathrm{rad}}(\omega)}\right|^2 R_{\mathrm{TFET}}. \tag{S9}$$

Supplementary Note 4.2: Bandstructure and charge density in bilayer graphene

BLG in external electric field is described by the Hamiltonian[S26,S27]

$$\hat{H}(\mathbf{k}) = \begin{pmatrix} -e\varphi_t & \hbar v_0(\pm k_x - ik_y) & 0 & 0 \\ \hbar v_0(\pm k_x + ik_y) & -e\varphi_t & \gamma_1 & 0 \\ 0 & \gamma_1 & -e\varphi_b & \hbar v_0(\pm k_x - ik_y) \\ 0 & 0 & \hbar v_0(\pm k_x + ik_y) & -e\varphi_b \end{pmatrix}, \tag{S10}$$

in the vicinity of K, K' points of the Brillouin zone, where φ_t, φ_b are the electric potentials at top and bottom graphene layers, $\gamma_1 = 0.38$ eV, $v_0 = 10^6$ m/s, and the signs depend on the valley.

The corresponding conduction and valence band dispersions are

$$\begin{aligned} E_{c,v}(k) &= -e\varphi_+ \pm E(k), \\ E(k) &= \sqrt{\frac{E_g^2}{4} + \left[\sqrt{\frac{\gamma_1^2 - E_g^2}{4} + (\hbar v_0 k)^2} - \frac{\gamma_1^2}{2\sqrt{\gamma_1^2 - E_g^2}}\right]^2} \end{aligned} \tag{S11}$$

with a bandgap

$$E_g(\varphi_-) = \frac{\gamma_1}{\sqrt{\gamma_1^2 + e^2\varphi_-^2}}|e\varphi_-|, \tag{S12}$$

where $\varphi_+ \equiv (\varphi_t + \varphi_b)/2$ is the average potential of graphene layers, and $\varphi_- \equiv \varphi_t - \varphi_b$ is the interlayer voltage. The bands have a "Mexican hat" shape with circular extrema around the corners of the Brillouin zone (Supplementary Figure 5).

The inverse dispersion relation is

$$k_\pm(E - e\varphi_+) = \frac{1}{\hbar v_0}\sqrt{E^2 + \frac{e^2\varphi_-^2}{4} \pm \sqrt{\left(\gamma_1^2 + e^2\varphi_-^2\right)\left(E^2 - \frac{E_g^2}{4}\right)}}. \tag{S13}$$

It is double-valued within the "Mexican hat" region, $E_g/2 < |E| \le |e\varphi_-|$, while only a single solution k_+ remains above the hat, $|E| > |e\varphi_-|$.

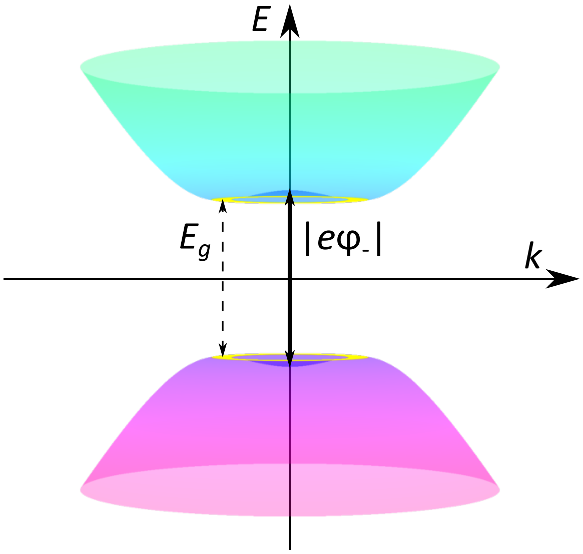

Supplementary Figure 5. **Bilayer graphene bandstructure.** Bandstructure of biased bilayer graphene described by Hamiltonian (S10) (only the conduction and valence bands are shown). Circular band extrema are highlighted in yellow.

Given the dispersion relation (S11), we can express the charge density $\rho_+ \equiv \rho_t + \rho_b$ in BLG at zero temperature through the chemical potential measured from the midgap, $\tilde{\mu} \equiv \mu + e\varphi_+$, and vice versa:

$$\rho_+(\tilde{\mu}) = \begin{cases} 0 & \text{if } |\tilde{\mu}| < \frac{E_g}{2}, \\ -e\frac{k_F^2}{\pi}\operatorname{sgn}\tilde{\mu}, k_F = k_+(\tilde{\mu}) & \text{if } |\tilde{\mu}| \geq \frac{|e\varphi_-|}{2}, \\ -e\frac{k_{F+}^2 - k_{F-}^2}{\pi}\operatorname{sgn}\tilde{\mu}, k_{F\pm} = k_\pm(\tilde{\mu}) & \text{if } \frac{E_g}{2} < |\tilde{\mu}| < \frac{|e\varphi_-|}{2}, \end{cases}$$
$$\tilde{\mu}(\rho_+) = \begin{cases} -E(k_F)\operatorname{sgn}\rho_+, k_F = \sqrt{\frac{\pi|\rho_+|}{e}} & \text{if } |\rho_+| \geq \rho_{\text{hat}}, \\ -\sqrt{\frac{E_g^2}{4} + \frac{1}{4\left(\gamma_1^2 + e^2\varphi_-^2\right)}\left(\hbar v_0\sqrt{\frac{\pi|\rho_+|}{e}}\right)^4}\operatorname{sgn}\rho_+ & \text{if } 0 < |\rho_+| < \rho_{\text{hat}} \end{cases} \tag{S14}$$

where $\rho_{\text{hat}} = (e/\pi)(e\varphi_-/\hbar v_0)^2$ is the charge density corresponding to $\tilde{\mu} = \pm e\varphi_-/2$ (the Fermi level positioned at the tip of the "Mexican hat"). We have taken into account the double valley degeneracy in BLG.

Supplementary Note 4.3: Electrostatics of double-gated bilayer graphene

To calculate the band diagram of our TFET, we seek approximate analytical solution of electrostatic equations for double-gated BLG.

Let V_{tg}, V_{bg} be the potentials of the top and bottom gate, d_t, d_b the thicknesses of dielectric layers separating BLG from the gates, κ_t, κ_b the dielectric constants of these layers, and d the interlayer distance in BLG. Then, the total charge density $\rho_+ \equiv \rho_t + \rho_b$ and interlayer charge transfer $\rho_- \equiv (\rho_t - \rho_b)/2$ are related to the electric potentials φ_t, φ_b of top and bottom graphene layers by

$$\rho_+ = -C_t(V_{tg} - \varphi_t) - C_b(V_{bg} - \varphi_b),$$
$$\rho_- = -\frac{C_t(V_{tg} - \varphi_t)}{2} + \frac{C_b(V_{bg} - \varphi_b)}{2} + C_-^{\text{cl}}(\varphi_t - \varphi_b), \tag{S15}$$

where we introduced capacitances per unit area $C_-^{\text{cl}} \equiv \epsilon_0/d$, $C_t \equiv \kappa_t\epsilon_0/d_t$, $C_b \equiv \kappa_b\epsilon_0/d_b$.

The potentials of graphene layers stay close to the Fermi level (compared to the gate voltages), and we can substitute $V_{t/b} - \varphi_{t/b} \to V_{t/b} + \mu/e$ in the second equation. We cannot do the same in the first equation, otherwise it would not work properly in the undoped case. Instead, in the first equation we approximate $\varphi_t \approx \varphi_b \approx \varphi_+$ to decouple ρ_+, φ_+ and ρ_-, φ_-:

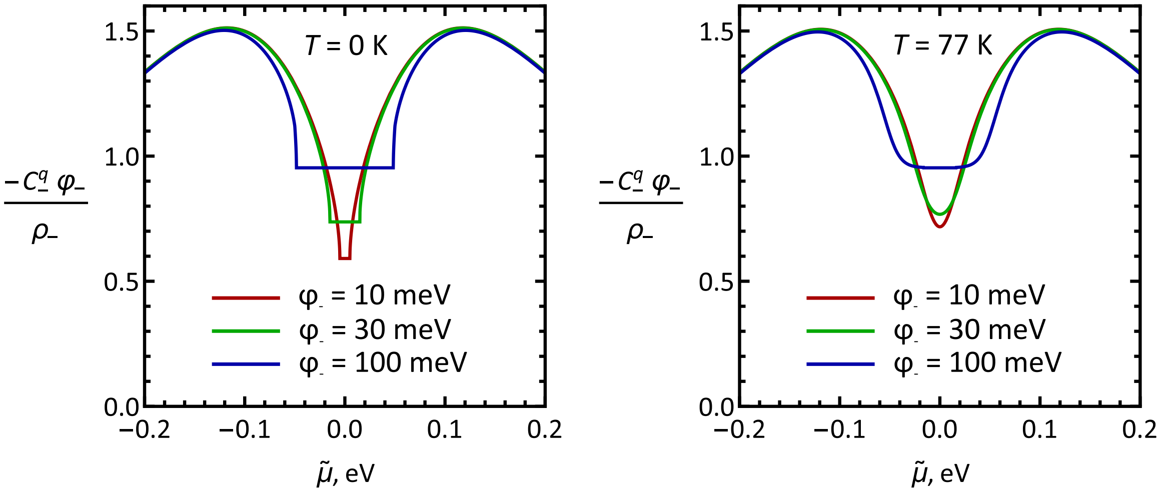

Supplementary Figure 6. Accuracy of the constant interlayer quantum capacitance approximation. Ratio between the interlayer charge transfer calculated using a constant interlayer quantum capacitance $C_-^q = 3e^2\gamma_1/(4\pi\hbar^2 v_0^2)$ and the exact interlayer charge transfer ρ_- calculated from Hamiltonian (S10) as described in[S26]. Left panel: zero temperature, right panel: $T = 77$ K.

$$\begin{aligned} \rho_+ &\approx -C_t V_{tg} - C_b V_{bg} + (C_t + C_b)\,\varphi_+, \\ \rho_- &= -\frac{C_t\,(V_{tg} + \mu/e)}{2} + \frac{C_b\,(V_{bg} + \mu/e)}{2} + C_-^{\rm cl}\varphi_-. \end{aligned} \tag{S16}$$

The introduced approximations essentially amount to a minor shift of gate voltages, by the order of magnitude equal to φ_t, φ_b.

These equations have to be supplemented with explicit expressions for $\rho_\pm(\tilde{\mu}, \varphi_-)$ in BLG ($\tilde{\mu} \equiv \mu + e\varphi_+$ is the chemical potential with respect to the midgap). To facilitate analytical treatment, we use zero-temperature expression for the total charge density (S14) and a constant quantum capacitance model for the interlayer charge transfer:

$$\begin{aligned} \rho_+ &= \begin{cases} 0 & \text{if } |\tilde{\mu}| \le \frac{E_g(\varphi_-)}{2}, \\ \rho_+(\tilde{\mu}) & \text{if } |\tilde{\mu}| > \frac{E_g(\varphi_-)}{2}, \end{cases} \\ \rho_- &\approx -C_-^q \varphi_-. \end{aligned} \tag{S17}$$

The constant interlayer quantum capacitance $C_-^q = 3e^2\gamma_1/(4\pi\hbar^2 v_0^2)$ approximates the interlayer charge transfer in BLG over a wide range of bangaps and doping levels within 50% accuracy (see Supplementary Figure 6).

Now, the equation for ρ_- becomes trivial to solve, while the equation for ρ_+ requires some additional simplifications to allow analytical solution. We consider two opposite cases: (1) Fermi level lies within the bandgap, (2) Fermi level lies outside the gap. In the first case, $\rho_+ = 0$ at zero temperature, and φ_+ is readily obtained from (S16). In the second case, we can pick some initial guess for φ_+, find ρ_+ from (S16), and find a better approximation for φ_+ from (S17). Since the quantum capacitance $C_+^q \sim \epsilon_0/d$ is much larger than $C_t + C_b$, the precise value of the initial guess is unimportant, and we initially assume the Fermi level is pinned at the band edge, $\tilde{\mu} = \pm E_g/2$ (this choice avoids spurious discontinuities in $\varphi_+(V_{tg}, V_{bg})$).

The overall procedure is summarized in the following equations:

$$\begin{aligned} \varphi_- &\approx \frac{C_t\,(V_{tg} + \mu/e) - C_b\,(V_{bg} + \mu/e)}{2C_-}, C_- \equiv C_-^{\rm cl} + C_-^q, \\ E_g &= E_g(\varphi_-), \\ \tilde{\mu}_0 &= e\frac{C_t\,(V_{tg} + \mu/e) + C_b\,(V_{bg} + \mu/e)}{C_t + C_b}, \\ \tilde{\mu} &\approx \begin{cases} \tilde{\mu}_0 & \text{if } |\tilde{\mu}_0| \le \frac{E_g}{2}, \\ \tilde{\mu}(\rho_+),\, \rho_+ = -\frac{C_t + C_b}{e}\left(\tilde{\mu}_0 - \frac{E_g}{2}\,\mathrm{sgn}\,\tilde{\mu}_0\right) & \text{if } |\tilde{\mu}_0| > \frac{E_g}{2}, \end{cases} \end{aligned} \tag{S18}$$

where $E_g(\varphi_-)$, $\tilde{\mu}(\rho_+)$ are given by (S12) and (S14).

In our calculations, we used $d_t = 80$ nm, $d_b = 50$ nm, $d = 0.335$ nm, and $\kappa_t = \kappa_b = 3.76$ (out-of-plane static dielectric constant of hexagonal boron nitride[S28]).

We use this parallel-plate capacitor model to find the electric potentials φ_{+S}, φ_{+C} and interlayer voltages φ_{-S}, φ_{-C} in the source region and in the channel in absence of ac signal, and also to calculate the channel response to an ac signal, see Supplementary Note 4.6. In the source region, there is only the bottom gate, while the role of a top gate is played by infinity, held at zero potential. This means $C_t = 0$, and the top gate disappears from the equations.

Supplementary Note 4.4: Tunneling field

In a TFET based on double-gated BLG, a tunnel juntion is formed under the top gate edge, where the parallel-plate capacitor model of Supplementary Note 4.3 cannot be applied, and an accurate calculation of the tunneling field requires solving a two-dimensional electrostatic problem. This problem can be solved analytically in the absence of BLG[S29], and the answer is

$$\tilde{E}_x = \frac{1-\tilde{\varphi}}{\left[1+\tilde{y}\left(1-\tilde{\varphi}\right)\cot\tilde{y}\tilde{\varphi}\right]^2+\tilde{y}^2\left(1-\tilde{\varphi}\right)^2}, \tag{S19}$$

where

$$\tilde{E}_x \equiv \frac{E_x d_{tb}}{\pi\left[\varphi\left(x=-\infty\right)-\varphi\left(x=+\infty\right)\right]}, \tilde{y} \equiv \frac{\pi y}{d_{tb}}, \tilde{\varphi} \equiv \frac{\varphi-\varphi\left(x=-\infty\right)}{\varphi\left(x=+\infty\right)-\varphi\left(x=-\infty\right)} \tag{S20}$$

are the dimensionless field in the plane of BLG, dimensionless position of BLG with respect to the gates ($\tilde{y} = 0$ at the bottom gate and π at the top gate), and dimensionless electric potential at the point where the field is calculated. $d_{tb} = d_t + d_b + d$ is the distance between gates, $\varphi(x=-\infty) = V_{bg}$ and $\varphi(x=+\infty) = V_{bg} + (y/d_{tb})(V_{tg} - V_{bg})$ are the electric potential in the source region and in the channel. The top and bottom dielectrics are assumed to be the same, as in our experiment.

Across a wide range of $\tilde{y}$, $\tilde{E}_x$ is close to its low-$\tilde{y}$ limit

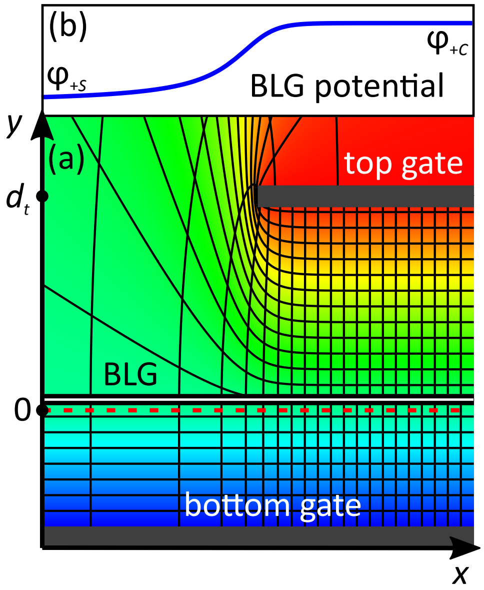

Supplementary Figure 7. Electric field in the tunnel junction. a Color map showing the distribution of electric potential near the source-channel junction. Black lines: equipotential lines and field lines. Dashed red line: fictitious conductor introduced to obtain the correct potential in the source and the channel without explicitly considering screening by BLG. Potential above the fictitious conductor was calculated as prescribed in Ref. S29, potential below the fictitious conductor was calculated in the parallel-plate capacitor model. **b** Electric potential $\varphi_+(x)$ inside BLG.

$$\tilde{E}_x \approx \tilde{\varphi}^2 \left(1-\tilde{\varphi}\right). \tag{S21}$$

In the presence of BLG, exact calculation of the tunneling field would require solving the two-dimensional electrostatic problem numerically. To avoid this, we notice that adding BLG into the system reduces $\varphi\left(x=+\infty\right)-\varphi\left(x=-\infty\right)$ from several volts to tens or hundreds of millivolts. This suggests to approximate the screening by BLG via introducing a fictitious perfect conductor placed very close to the BLG. The potential of this conductor and its distance from the BLG are chosen so as to reproduce the correct potentials in the source and channel regions of BLG.

The resulting electric potential distribution in the system is shown in Supplementary Figure 7. Introducing the fictitious conductor allows us to keep Eq. (S21) for the electric field in BLG, if d_{tb} is replaced with d_t in the definition of (Eq. (S20)), and $\varphi\left(x=\pm\infty\right)$ are calculated in the parallel-plate capacitor model described in Supplementary Note 4.3.

Knowing the distribution of electric potential in BLG, we can calculate the tunnel current though the source-channel junction. Before we actually do this, we introduce two additional simplifications. First, we neglect field variations inside the barrier and assume tunneling through uniform field. This field is calculated at the point where the tunneling electron crosses the midgap ($E+e\varphi=0$, where E is the electron energy). Second, instead of using different values of the tunneling field for electrons of different energies, we use a single value calculated for energy $E=(E_{\text{tun,min}}+E_{\text{tun,max}})/2$. $E_{\text{tun,min}}$ and $E_{\text{tun,max}}$ are the boundaries of the energy region where tunneling is possible. Assuming zero temperature and both quasi-Femi levels μ_S, μ_C in the source and the channel (near its beginning) lying within the band overlap region, we can write $E_{\text{tun,min}}=\min\left\{\mu_S,\mu_C\right\}$ and $E_{\text{tun,max}}=\max\left\{\mu_S,\mu_C\right\}$. (Remember that we are interested in the small-signal case, when the quasi-Fermi levels are close to each other and either lie both inside the band overlap region, or both outside. In the latter case, tunneling is impossible.)

To summarize, we use the following expression for the tunneling field:

$$F_{\text{tun}} \approx \frac{\pi\left|\varphi_{+C}-\varphi_{+,\text{tun}}\right|}{d_t}\left(\frac{\varphi_{+,\text{tun}}-\varphi_{+S}}{\varphi_{+C}-\varphi_{+S}}\right)^2, \tag{S22}$$

where φ_{+S}, φ_{+C} are calculated as described in Supplementary Note 4.3, and $-e\varphi_{+,\text{tun}}=\left(\mu_S+\mu_C\right)/2$.

Supplementary Note 4.5: Responsivity of the source-channel junction

A zero-temperature ballistic expression for the tunnel current through the source-channel junction is

$$I_{\leftarrow s,\text{tun}} = 8eW\int_{\mu_C}^{\mu_S}\frac{dE}{2\pi\hbar}\int_{-k_{\perp\max}(E)}^{k_{\perp\max}(E)}\frac{dk_\perp}{2\pi}\mathcal{D}\left(E,k_\perp\right) \tag{S23}$$

if $\mu_S>\mu_C$ (the opposite case is treated similarly). Here, $W=6.2$ μm is the channel width, $\mathcal{D}\left(E,k_\perp\right)$ is the barrier transparency, the wavevector integral is taken up to the maximum possible transverse wavevector $k_{\perp\max}(E)$ that an electron with energy E can have both in the source and in the channel, and the factor of 8 results from two spin projections, two valleys, and two tunneling paths in the imaginary k-space (interference between them[S30] is neglected).

An analytical approximation can be derived by expanding the WKB barrier transparency in powers of $k_\perp$ up to second order and extending the wavevector integration up to infinity[S31]:

$$\begin{aligned} I_{\leftarrow s,\text{tun}} &\approx \frac{2e}{\pi^{3/2}\hbar}\mathcal{D}_{\text{tun}}k_{\perp\text{tun}}W\left(\mu_S-\mu_C\right), \\ \mathcal{D}_{\text{tun}} &\approx \exp\left(-\frac{\pi\sqrt{\gamma_1 E_{g,\text{tun}}^3}}{4\hbar v_0 e F_{\text{tun}}}\right), \\ k_{\perp\text{tun}} &\approx \sqrt{\frac{4}{\pi}\sqrt{\frac{\gamma_1}{E_{g,\text{tun}}}}\frac{eF_{\text{tun}}}{\hbar v_0}}. \end{aligned} \tag{S24}$$

We assume that the transition between the source and the channel has the same shape for both the interlayer voltage and the electric potential and, therefore, the tunnel current flows through the bandgap $E_{g,\text{tun}}\approx\left|\varphi_{-,\text{tun}}\right|$, where

$$\frac{\varphi_{-,\text{tun}}-\varphi_{-S}}{\varphi_{-C}-\varphi_{-S}} = \frac{\varphi_{+,\text{tun}}-\varphi_{+S}}{\varphi_{+C}-\varphi_{+S}}. \tag{S25}$$

At experimental conditions, the bandgap does not exceed 60 meV, so we use $E_g \approx |\varphi_-|$ instead of a more accurate expression (S12).

Expressions (S23), (S24) require that the chemical potentials μ_S, μ_C are taken at the points where the deviations of the carrier distributions from the Fermi-Dirac form are negligible. Since we consider the tunnel junction connected in series with the channel, we need an expression for the tunnel current in terms of the voltage *directly at the junction*, otherwise a certain part of the channel would be counted twice. This can be achieved by introducing a $1 - \mathcal{D}_{\rm tun}$ correction in the denominator:

$$I_{\leftarrow s,{\rm tun}} \approx \frac{2e}{\pi^{3/2}\hbar}\frac{\mathcal{D}_{\rm tun}}{1-\mathcal{D}_{\rm tun}} k_{\perp{\rm tun}} W\left(\mu_S - \mu_C\right), \tag{S26}$$

similarly to the one-dimensional Landauer formula containing $\mathcal{D}/(1-\mathcal{D})$[S32,S33].

The idealistic model that led to Eq. (S26) gives very small barrier transparency and huge tunnel resistance, orders of magnitude larger than in our experiment. This suggests there is some mechanism affecting the junction resistance, most likely electron-hole puddles, that create field fluctuations and may increase the average tunneling field. We take this effect into account phenomenologically, introducing a single fitting parameter $F_{\rm fluct}$, which represents the average fluctuating field and is added to the tunneling field (S22) calculated without disorder:

$$\begin{aligned} I_{\leftarrow s,{\rm tun}} &\approx \frac{2e}{\pi^{3/2}\hbar}\frac{\mathcal{D}_{\rm tun}}{1-\mathcal{D}_{\rm tun}} k_{\perp{\rm tun}} W\left(\mu_S - \mu_C\right), \\ \mathcal{D}_{\rm tun} &\approx \exp\left(-\frac{\pi\sqrt{\gamma_1 E_{g,{\rm tun}}^3}}{4\hbar v_0 e\left(F_{\rm tun}+F_{\rm fluct}\right)}\right), \\ k_{\perp{\rm tun}} &\approx \sqrt{\frac{4}{\pi}\sqrt{\frac{\gamma_1}{E_{g,{\rm tun}}}}\frac{e\left(F_{\rm tun}+F_{\rm fluct}\right)}{\hbar v_0}}. \end{aligned} \tag{S27}$$

This is the final expression for the tunnel current that we used in our calculations. The value $F_{\rm fluct} = 8$ kV/cm was found by fitting the experimental resistance in the tunnel regime and simultaneously gave responsivity in reasonable agreement with the experiment.

Assuming grounded source, $\mu_S = 0$, we identify μ_C with $-eV_{sS}$ and V_{tg} with V_{GS} from Supplementary Note 4.1. Now, we can calculate the junction conductance as $G_S = -e\partial I_{\leftarrow s,{\rm tun}}/\partial\mu_C$ and the intrinsic tunnel junction and tunnel-gate responsivities through (S6). When the doping types of source and channel are the same, or channel is undoped, there is no *tunnel* junction. In this case, we set the junction conductance to infinity and tunnel junction and tunnel-gate responsivities to zero.

Supplementary Note 4.6: Responsivity of a long double-gated channel

In this section, we consider resistive self-mixing in a long[S34] *double-gated* channel and find its responsivity. Our derivation closely follows that of Ref. S24, but extends it by (1) allowing the carrier density to depend separately on the top gate voltage and the Fermi level (because $\rho_+ = \rho_+\left(\mu + eV_{tg}\right)$ is no longer true in the presence of a bottom gate), (2) using frequency-dependent channel conductivity.

The basic assumptions of our model are that the dc channel conductivity $\sigma_{\rm dc}(x,t)$ is instantaneously related to the local charge density $\rho_+(x,t)$, which, in turn, is related (also locally and instantaneously) to the top gate voltage $V_{tg}(t)$ and the Fermi level $\mu(x,t)$. Response to ac perturbations is described within the Drude model. Together with the charge conservation, we get a system of four equations:

$$\begin{aligned} \rho_+(x,t) &= \rho_+\left(V_{tg}(t), \mu(x,t)\right), \\ \frac{\partial J_{\leftarrow}(x,t)}{\partial t} &= -\frac{1}{e}\frac{\sigma_{\rm dc}(x,t)}{\tau}\frac{\partial\mu(x,t)}{\partial x} - \frac{J_{\leftarrow}(x,t)}{\tau}, \\ \sigma_{\rm dc}(x,t) &= \sigma_{\rm dc}\left[\rho_+(x,t)\right], \\ \frac{\partial\rho_+(x,t)}{\partial t} &= \frac{\partial J_{\leftarrow}(x,t)}{\partial x}, \end{aligned} \tag{S28}$$

with the boundary conditions of grounded source and zero drain current:

$$\mu(0,t) = 0, J_{\leftarrow}(+\infty,t) = 0. \tag{S29}$$

The top gate voltage consists of a constant bias and an ac signal, $V_{tg}(t) = V_{tg}^{(0)} + V_{\rm in}\cos(\omega t)$. (Hereafter, quantities in the absence of the ac signal will be denoted by the $^{(0)}$ superscript, while next orders in $V_{\rm in}$ will be denoted by $^{(1)}$ and $^{(2)}$, as in Supplementary Note 4.1.)

To the first order in $V_{\rm in}$, we obtain

$$\begin{aligned}
\rho_+^{(1)} &= \frac{\partial \rho_+}{\partial V_{tg}} V_{\rm in} + \frac{\partial \rho_+}{\partial \mu}\mu^{(1)}, \\
J_{\leftarrow}^{(1)} &= -\frac{1}{e}\frac{\sigma_{\rm dc}^{(0)}}{1 - i\omega\tau}\frac{\partial \mu^{(1)}}{\partial x}, \\
\sigma_{\rm dc}^{(1)} &= \frac{d\sigma_{\rm dc}}{d\rho_+}\rho_+^{(1)}, \\
-i\omega\rho_+^{(1)} &= \frac{\partial J_{\leftarrow}^{(1)}}{\partial x}.
\end{aligned} \tag{S30}$$

Using the boundary conditions (S29), we get the following solution

$$\begin{aligned}
\mu^{(1)}(x) &= -\left(\frac{\partial \rho_+}{\partial \mu}\right)^{-1}\frac{\partial \rho_+}{\partial V_{tg}} V_{\rm in}\left[1 - e^{iq_{\rm pl}x}\right], \\
\rho_+^{(1)}(x) &= \frac{\partial \rho_+}{\partial V_{tg}} V_{\rm in} e^{iq_{\rm pl}x}, \\
J_{\leftarrow}^{(1)}(x) &= -\frac{1}{e}\frac{\sigma_{\rm dc}^{(0)}}{1 - i\omega\tau} iq_{\rm pl}\left(\frac{\partial \rho_+}{\partial \mu}\right)^{-1}\frac{\partial \rho_+}{\partial V_{tg}} V_{\rm in} e^{iq_{\rm pl}x}, \\
q_{\rm pl} &\equiv \sqrt{\frac{i\omega(1 - i\omega\tau)}{\sigma_{\rm dc}^{(0)}}\frac{\partial \rho_+}{\partial(-\mu/e)}}.
\end{aligned} \tag{S31}$$

Having found the first-order current, we can write the channel "source-gate" conductance (W is the channel width):

$$\tilde{G}_{\rm ch}(\omega) \equiv \frac{J_{\leftarrow}^{(1)}(0)W}{V_{\rm in}} = -\frac{\sigma_{\rm dc}^{(0)}}{1 - i\omega\tau} iq_{\rm pl} W \left(\frac{\partial(-\mu/e)}{\partial V_{tg}}\right)_{\rho_+}. \tag{S32}$$

(Note that we use $\exp(-i\omega t)$ for the time dependence of harmonic signals instead of $\exp(+j\omega t)$ convention prevalent in electrical engineering, resulting in reactances having unconventional signs.)

The equation on the second order dc current results from the zero dc drain current condition,

$$J_{\leftarrow}^{(2)} = -\frac{1}{e}\sigma_{\rm dc}^{(0)}\frac{\partial \mu^{(2)}}{\partial x} - \frac{1}{2e}\,{\rm Re}\left(\sigma_{\rm dc}^{(1)}\frac{\partial \mu^{(1)*}}{\partial x}\right) = 0, \tag{S33}$$

yielding the intrinsic channel responsivity

$$\begin{aligned}
R_{{\rm ch},i} \equiv \frac{V_{\rm out}}{|V_{\rm in}|^2/2} &\equiv \frac{\left[\mu^{(2)}(+\infty) - \mu^{(2)}(0)\right]/(-e)}{|V_{\rm in}|^2/2} \\
&= -\frac{1}{2}\left(\frac{\partial \ln\sigma_{\rm dc}}{\partial V_{tg}}\right)_{\mu}\left(\frac{\partial(-\mu/e)}{\partial V_{tg}}\right)_{\rho_+}.
\end{aligned} \tag{S34}$$

The expressions (S32), (S34), and the definition of $q_{\rm pl}$ (S31) differ from the results of Ref. S24 by two extra factors. The first factor $(\partial(-\mu/e)/\partial V_{tg})_{\rho_+}$ is unity in a single-gated FET and reduces to approximately $C_t/(C_t + C_b) = \tilde{d}_b/(\tilde{d}_t + \tilde{d}_b)$ in the presence of a bottom gate. The second factor $1/(1 - i\omega\tau)$ appears due to the frequency dependence of conductivity.

Calculations show that the difference between $\left(\partial\left(-\mu/e\right)/\partial V_{tg}\right)_{\rho_+}$ and $\tilde{d}_b/(\tilde{d}_t+\tilde{d}_b)$ is minor and can be neglected within the accuracy of our model, so we used the following expressions for the channel "source-gate" conductance and intrinsic channel responsivity:

$$\begin{aligned} \tilde{G}_{\rm ch}(\omega) &= -\frac{\sigma_{\rm dc}^{(0)}}{1-i\omega\tau} i q_{\rm pl} W \frac{\tilde{d}_b}{\tilde{d}_t+\tilde{d}_b}, \\ R_{{\rm ch},i} &= -\frac{1}{2}\left(\frac{\partial \ln\sigma_{\rm dc}}{\partial V_{tg}}\right)_{\mu} \frac{\tilde{d}_b}{\tilde{d}_t+\tilde{d}_b}, \\ q_{\rm pl} &\equiv \sqrt{\frac{i\omega(1-i\omega\tau)}{\sigma_{\rm dc}^{(0)}}\frac{\partial\rho_+}{\partial\left(-\mu/e\right)}}. \end{aligned} \tag{S35}$$

The derivatives in (S35) were evaluated with the help of the approximate electrostatic model presented in Supplementary Note 4.3 and the constant-mobility approximation for the channel dc conductivity:

$$\sigma_{\rm dc}^{(0)} = \left|\rho_+\right| \mu_{\rm BLG} + \sigma_{\rm residual}(V_{bg}), \tag{S36}$$

where we take $\mu_{\rm BLG} = 10^5$ cm^2/(V·s) (according to measurements performed on similar devices[S1]). The transport relaxation time τ was taken to be 2 ps according to the relation $\mu_{\rm BLG} = e\tau/m^*$, where $m^* = \gamma_1/2v_0^2$ (this is the carrier effective mass in the band extrema of gapless BLG; in gapped BLG band dispersion is similar to the gapless case except in close vicinity of the band edges, so we neglect the bandgap dependence of m^*).

The residual conductivity $\sigma_{\rm residual}$ due to potential fluctuations in the channel was obtained by fitting the following formula to the experimental dc resistance at the channel neutrality point:

$$\frac{L}{W}\sigma_{\rm residual}^{-1}(V_{bg}) = \frac{r_\infty V_{bg}^2 + r_0 V_0^2}{V_{bg}^2+V_0^2}. \tag{S37}$$

The fitting procedure yielded $r_0 = 200\ \Omega$, $r_\infty = 150$ kΩ, $V_0 = 5.5$ V.

Using the intrinsic channel responsivity and channel "source-gate" conductance, together with the intrinsic tunnel junction and tunnel-gate responsivities and the tunnel junction conductance found in Supplementary Note 4.5, we can obtain the total responsivity of our transistor through Eq. (S5) and convert it to the photodetector responsivity through Eq. (S9).

Supplementary Note 5: Performance limits of BLG TFET photodetectors

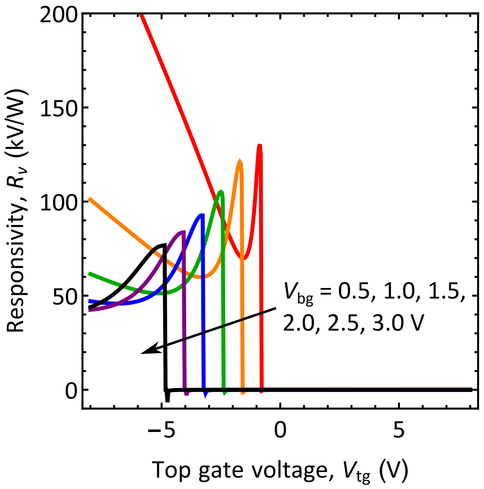

Supplementary Figure 8. **Responsivity of an ideal device.** Calculated responsivity of our photodetector in absence of electric potential fluctuations.

The theory described in the previous Supplementary Sections was used to calculate the theoretical responsivity of our photodetector, which is shown in Fig. 4 of the main text. The detector responsivity in our theory is limited by the electric potential fluctuations and could be substantially improved in devices with reduced density of charged impurities. Supplementary Figure 8 shows the theoretical responsivity of our photodetector in absence of potential fluctuations (that is, with $F_{\text{fluct}} = 0$), which reaches hundreds kV/W.

Another way to increase detector responsivity is to exploit large nonlinearity of the tunnel junction at small values of band overlap (when the tunnel current is about to be switched off). This requires that the conduction band edge in the source region is simultaneously aligned with the valence band edge in the channel (or vice versa) and with the Fermi level.

Such kind of band alignment can be realized by introducing an additional gate above the source region and could potentially result in a very large nonlinearity even at room temperature[S31], which would yield infinite responsivity in the idealized model (no potential fluctuations, no leakage currents). This is easy to show by considering a power-law dependence of the tunnel conductance on the gate voltage, $G_S \propto (V_{tg} - V_{\text{th}})^\alpha$ for $V_{tg} > V_{\text{th}}$ and zero otherwise, which results from the power-law dispersion $k(E)$ near the band edges. Taking the logarithmic derivative of G_S with respect to the gate voltage, we obtain $|R_{\text{TG},i}| = \alpha/(V_{tg} - V_{\text{th}})$ for nonzero α, or a δ-peak at $V_{tg} = V_{\text{th}}$ for $\alpha = 0$. A similar argument holds for $|R_{\text{TJ},i}|$, since the band alignment is affected not only by the gate voltage, but also by the Fermi level in the channel.

In practice, the maximum achievable responsivity will be limited by potential fluctuations and leakage currents. Thermionic leakage hinders the performance of our detector at non-cryogenic temperatures because of the small bandgap (< 60 meV) realized in our TFET, but this problem can be mitigated by increasing the bandgap, either by applying a larger vertical field to BLG, or by using larger-gap materials, such as black phosphorus. Electric potential fluctiations present a more fundamental issue and limit the logarithmic derivatives of the tunnel conductance to $\sim 1/V_{\text{fluct}}$, where V_{fluct} is the magnitude of these fluctuations.

Assuming the total responsivity is dominated by R_{TJ} (as in our photodetector) and using equations (S5), (S6), and (S9), we can estimate the achievable room-temperature noise equivalent power as

$$\begin{aligned}\text{NEP}_{\min} = \frac{\sqrt{4 r_{2\text{pt}} k_B T}}{|R_{\text{v}}|} = \frac{\sqrt{4 r_{2\text{pt}} k_B T}}{4 Z_{\text{rad}} \left| \frac{Z_{\text{GS}}}{Z_{\text{GS}} + Z_{\text{rad}}} \right|^2 |R_{\text{TFET}}|} \approx \frac{\sqrt{4 r_S k_B T}}{4 Z_{\text{rad}} \left(\frac{r_S}{r_S + Z_{\text{rad}}} \right)^2 \frac{1}{2} \left| \frac{\partial \ln G_S}{\partial V_{sS}} \right|} \\ \approx \frac{16}{9} \sqrt{3}\, V_{\text{fluct}} \sqrt{\frac{k_B T}{Z_{\text{rad}}}},\end{aligned} \tag{S38}$$

where $r_S = G_S^{-1}$ is the resistance of the source tunnel junction. To minimize the noise equivalent power, we assumed $r_S = 3 Z_{\text{rad}}$, the drain junction is absent, and the channel resistance is negligible.

Taking $Z_{\text{rad}} = 75\ \Omega$ and $V_{\text{fluct}} = 1$ mV (an experimentally achievable value[S35]), we estimate that the room-temperature noise equivalent power in TFET-based photodetectors can be made as low as 0.02 pW/$\sqrt{\text{Hz}}$ (shown in

Supplementary Figure 3).

SUPPLEMENTARY REFERENCES

S1. Bandurin, D. A. *et al.* Resonant terahertz detection using graphene plasmons. *Nature Communications* **9**, 1–8 (2018).
S2. SCONTEL. http://www.scontel.ru.
S3. QMC Instruments Ltd. http://www.terahertz.co.uk.
S4. Infrared Laboratories, Inc. https://www.infraredlaboratories.com.
S5. Alsop, D. C., Inman, C., Lange, A. E. & Wilbanks, T. Design and construction of high-sensitivity, infrared bolometers for operation at 300 mK. *Applied Optics* **31**, 6610–6615 (1992).
S6. Tanaka, S. T. *et al.* 100-mK bolometric receiver for low-background astronomy. In *Infrared Detectors and Instrumentation*, vol. 1946, 110–115 (International Society for Optics and Photonics, 1993).
S7. Hubmayr, J. *et al.* Photon-noise limited sensitivity in titanium nitride kinetic inductance detectors. *Applied Physics Letters* **106**, 073505 (2015).
S8. Monfardini, A. *et al.* A dual-band millimeter-wave kinetic inductance camera for the IRAM 30 m telescope. *The Astrophysical Journal Supplement Series* **194**, 24 (2011).
S9. Kleinschmidt, P. *et al.* A highly sensitive detector for radiation in the terahertz region. *IEEE Transactions On Instrumentation and Measurement* **56**, 463–467 (2007).
S10. Dai, J.-H., Lee, J.-H., Lin, Y.-L. & Lee, S.-C. In(Ga)As quantum rings for terahertz detectors. *Japanese Journal of Applied Physics* **47**, 2924 (2008).
S11. Terasense Group, Inc. https://terasense.com/.
S12. Vicarelli, L. *et al.* Graphene field-effect transistors as room-temperature terahertz detectors. *Nature Materials* **11**, 865–871 (2012).
S13. Generalov, A. A., Andersson, M. A., Yang, X., Vorobiev, A. & Stake, J. A 400-GHz graphene FET detector. *IEEE Transactions on Terahertz Science and Technology* **7**, 614–616 (2017).
S14. Bandurin, D. A. *et al.* Dual origin of room temperature sub-terahertz photoresponse in graphene field effect transistors. *Appl. Phys. Lett.* **112**, 141101 (2018).
S15. Bauer, M. *et al.* A high-sensitivity AlGaN/GaN HEMT terahertz detector with integrated broadband bow-tie antenna. *IEEE Transactions on Terahertz Science and Technology* **9**, 430–444 (2019).
S16. Zagrajek, P. *et al.* Time resolution and dynamic range of field-effect transistor-based terahertz detectors. *Journal of Infrared, Millimeter, and Terahertz Waves* **40**, 703–719 (2019).
S17. FerrerasMayo, M., Cibiraite-Lukenskiene, D., Lisauskas, A., Grajal, J. & Krozer, V. Broadband sensing around 1 THz via a novel biquad-antenna-coupled low-NEP detector in CMOS. *IEEE Transactions on Terahertz Science and Technology* (2020).
S18. Qin, H. *et al.* Detection of incoherent terahertz light using antenna-coupled high-electron-mobility field-effect transistors. *Applied Physics Letters* **110**, 171109 (2017).
S19. Virginia Diodes, Inc. https://www.vadiodes.com/.
S20. ACST GmbH. https://acst.de/.
S21. Retzloff, S. A., Young, A. & Hesler, J. L. A 1.46 THz Schottky receiver at cryogenic temperatures. In *2014 39th International Conference on Infrared, Millimeter, and Terahertz Waves (IRMMW-THz)*, 1–2 (IEEE, 2014).
S22. Zhang, Z., Rajavel, R., Deelman, P. & Fay, P. Sub-Micron Area Heterojunction Backward Diode Millimeter-Wave Detectors With 0.18 $pW/Hz^{1/2}$ Noise Equivalent Power. *IEEE Microwave and Wireless Components Letters* **21**, 267–269 (2011).
S23. Rahman, S. M., Jiang, Z., Shams, M. I. B., Fay, P. & Liu, L. A G-band monolithically integrated quasi-optical zero-bias detector based on heterostructure backward diodes using submicrometer airbridges. *IEEE Transactions on Microwave Theory and Techniques* **66**, 2010–2017 (2017).
S24. Sakowicz, M. *et al.* Terahertz responsivity of field effect transistors versus their static channel conductivity and loading effects. *Journal of Applied Physics* **110**, 054512 (2011).
S25. Sanchez, A., Davis Jr, C. F., Liu, K. C. & Javan, A. The MOM tunneling diode: Theoretical estimate of its performance at microwave and infrared frequencies. *Journal of Applied Physics* **49**, 5270–5277 (1978).
S26. Castro, E. V. *et al.* Electronic properties of a biased graphene bilayer. *Journal of Physics: Condensed Matter* **22**, 175503 (2010).
S27. McCann, E. & Koshino, M. The electronic properties of bilayer graphene. *Reports on Progress in Physics* **76**, 056503 (2013).
S28. Laturia, A., Van de Put, M. L. & Vandenberghe, W. G. Dielectric properties of hexagonal boron nitride and transition metal dichalcogenides: from monolayer to bulk. *npj 2D Materials and Applications* **2**, 1–7 (2018).
S29. Maxwell, J. C. *A Treatise on Electricity and Magnetism*, vol. 1 (Clarendon press, 1873). Art. 202.
S30. Nandkishore, R. & Levitov, L. Common-path interference and oscillatory Zener tunneling in bilayer graphene p-n junctions. *Proceedings of the National Academy of Sciences* **108**, 14021–14025 (2011).
S31. Alymov, G., Vyurkov, V., Ryzhii, V. & Svintsov, D. Abrupt current switching in graphene bilayer tunnel transistors enabled by van Hove singularities. *Scientific Reports* **6**, 24654 (2016).
S32. Datta, S. *Electronic Transport in Mesoscopic Systems* (Cambridge university press, 1997).
S33. Strictly speaking, a proper averaging of $\mathcal{D}(E, k_\perp)$ is required in the two-dimensional case. This is not an easy task, especially because we need not only linear, but also quadratic response, and the result will depend on the precise form of scattering that drives the carrier distributions toward equilibrium. This would anyway exceed the accuracy of our model,

so we resort to this simple substitution. Its only role in our model is to provide smooth interpolation between the gapless case (unit transparency, there is essentially no tunnel junction, its conductance should become infinite), and the gapped case (small transparency, (S24) is applicable).

S34. Our use of the long-channel approximation is motivated by the fact that the experimentally obtained plasmon resonances in responsivity are not as prominent as they are in the finite-channel theory. This suggests there are additional plasmon damping mechanisms not taken into account in our model, like interband absorption or effects of electron viscosity. Instead of complicating the model by considering them explicitly, we take into account the extra plasmon damping approximately, not by using a shorter decay length, but by setting the channel length to infinity.

S35. Uwanno, T., Taniguchi, T., Watanabe, K. & Nagashio, K. Electrically inert h-BN/bilayer graphene interface in all-two-dimensional heterostructure field effect transistors. *ACS Applied Materials & Interfaces* **10**, 28780–28788 (2018).
